# Exploration of patients’ and healthcare professionals’ perspectives on kidney failure risk and the use of the kidney failure risk equation in MULTIPle lOng-term condItions aNd frailTy (MULTIPOINT) study: a qualitative interview and focus group study protocol

**DOI:** 10.1136/bmjopen-2024-085843

**Published:** 2024-10-18

**Authors:** Heather Walker, Michael K Sullivan, Bhautesh Dinesh Jani, Patrick B Mark, Katie I Gallacher

**Affiliations:** 1 School of Cardiovascular and Metabolic Health, University of Glasgow, Glasgow, UK; 2 Renal and Transplant Unit, NHS Greater Glasgow and Clyde, Queen Elizabeth University Hospital, Glasgow, UK; 3 General Practice and Primary Care, School of Health and Wellbeing, University of Glasgow, Glasgow, UK

**Keywords:** Frailty, Chronic renal failure, Prognosis, Qualitative research, Chronic Disease

## Abstract

**Abstract:**

**Introduction:**

Clinical guidelines recommend the use of the kidney failure risk equation (KFRE) to guide the referral of individuals with chronic kidney disease (CKD) to secondary kidney care services. People living with CKD frequently experience multiple long-term conditions (multimorbidity) and/or frailty. This may impact patients’ or carers’ perceptions of kidney failure in the context of other health problems and associated risks and emphasises the need for shared decision-making. This paper presents the research protocol for the exploration of patients’ and healthcare professionals’ perspectives on kidney failure risk and the use of the KFRE in the MULTIPle lOng-term condItions aNd frailTy study. This study aims to investigate patient and healthcare professionals’ perspectives and expectations of the use of KFRE in individuals with CKD and multimorbidity and/or frailty, with a focus on shared decision-making.

**Methods and analysis:**

Analysis of semistructured interviews with adults who have CKD and multimorbidity and/or frailty and focus groups with healthcare professionals (who are involved in caring for patients with CKD). Framework analysis, underpinned by normalisation process theory, will be used to develop codes and explore themes from the interviews and focus groups. Patient and public involvement has been pivotal to the study conceptualisation and will continue to be embedded throughout the study.

**Ethics and dissemination:**

The study protocol has undergone peer review by the NHS Greater Glasgow and Clyde Research and Innovation team and has been granted ethical approval in August 2023 by the NHS Health Research Authority following a favourable opinion from the West of Scotland Research Ethics Committee (REC) 3 (IRAS ID: 325848, REC reference: 23WS/0119, Protocol number GN22RE559).

The results of the research will be disseminated through peer-reviewed publications and conferences, as well as to patient and public involvement groups who have been involved in the study and through knowledge exchange events.

**STRENGTHS AND LIMITATIONS OF THIS STUDY**

- This study explores the use of a kidney failure prognostic model in the shared decision-making and clinical management of people with chronic kidney disease and multiple long-term health conditions and/or frailty, through rigorous methods underpinned by middle range theory (normalisation process theory).
- The qualitative interview and focus group approach will aid the understanding of patients’ and healthcare professionals’ perspectives on kidney failure risk in the context of multimorbidity and/or frailty.
- All aspects of the study have been developed in partnership with patient and public representatives.
- Individuals approaching the end of life, those unable to provide informed consent or those who do not speak English are excluded from the study, which limits the validation of our findings in these populations.
- Participants are recruited from a single region of Scotland, UK. This potentially may reduce the diversity of the study population.

## Introduction

 People with chronic kidney disease (CKD) are frequently impacted by multiple long-term health conditions, also referred to as multimorbidity.^1^ In addition, individuals with CKD are more likely to experience frailty than those without CKD.^2^ Frailty refers to an elevated risk of rapid health deterioration and reduced ability to recover from illness which is typically due to a diminished physiological capacity.^3 4^

An important risk faced by a small proportion of people with CKD is the progression to kidney failure requiring treatment with kidney replacement therapy (KRT), in the form of long-term dialysis or kidney transplantation. Accurate risk prediction of kidney failure in CKD is important to allow clinicians to provide personalised healthcare. The Kidney Disease: Improving Global Outcome (KDIGO) 2012 Clinical Practice Guideline for CKD^5^ and the 2021 National Institute for Health and Care Excellence (NICE) guidelines for management of CKD^6^ recommend using the four-variable Kidney Failure Risk Equation (KFRE),^7^ composed of age, sex, estimated glomerular filtration rate and urine albumin creatinine ratio. This can inform adults with CKD about their 5-year risk of need for KRT and it can help guide referral of adults with CKD for assessment from primary care to secondary care kidney specialists. Despite this recommendation, there remain barriers to KFRE being used for all people with CKD, with various possible reasons for this.^8^

Multimorbidity is associated with a heightened risk of mortality,^9^ higher healthcare service utilisation, polypharmacy, high treatment burden and reduced quality of life.^10^ Treatment of single diseases, compared with holistic and generalised care approaches, can exacerbate these problems.^11^ Multimorbidity, frailty and associated factors such as treatment burden may influence how individuals wish information about kidney failure risk, assessment and management be communicated to them and how they make decisions around future care. Shared decision-making (SDM) can play an important role in helping individuals reach health-related decisions unique to their circumstances, preferences and priorities. SDM has been defined as ‘an approach where clinicians and patients share the best available evidence when faced with the task of making decisions, and where patients are supported to consider options, to achieve informed preferences’.^12^ Its utilisation in people with multiple long-term conditions may result in improved understanding of risks and benefits, enhance communication between healthcare professionals (HCPs) and patients and reduce difficulties around decision-making.^13 14^

To date, there has been limited research on communication and SDM in the CKD population, with a focus mainly on KRT decisions.^15^,^20^ More recently, there has been an acknowledgement that SDM is likely to be beneficial and preferred by patients with CKD, in decision-making scenarios.^21^ However, there is a lack of high-quality studies relating to the impact of SDM on clinical outcomes.^20^ A recent scoping review assessing interventions that support SDM in advanced kidney disease highlighted prognostic tools, including KFRE, that may be used to support treatment modality decisions.^19^ It is unclear how best to communicate the risk of kidney failure in patients with CKD who are frail and/or multimorbid or how SDM should be used when using prognostic tools such as KFRE. A small number of studies have examined the patient, family and HCP perspective on the use of the KFRE for a risk-based approach to guiding care.^22 23^ However, there is a paucity of research exploring the use of KFRE in more complex populations such as those with multimorbidity or frailty.

Despite this lack of research in the CKD population, decision-making about treatments has been studied in primary care populations, with a recent systematic review focusing on patients in primary care with complex care needs, identifying the types of decisional needs across a range of settings and contexts^24^ and demonstrating that SDM was associated with positive outcomes. Within this study, it was acknowledged that decisions concerning areas other than treatment options have been under-researched and warrant further consideration. A recent qualitative study examined the experience of decision-making from the perspective of older adults with multimorbidity and general practitioners (GPs).^25^ Themes identified from this study included the barrier of clinical uncertainty in communication and SDM and the difficulty of applying single organ or disease-specific guidelines in patients with multimorbidity, which may be applicable to implementing the KFRE in patients with multimorbidity and/or frailty.

Current literature does not address the use of SDM in the context of decisions relating to communication of the risk of kidney failure, referral to secondary care kidney specialists or the use of risk prediction models (such as KFRE) in making healthcare-related decisions in this context. Moreover, there is a lack of understanding around SDM in individuals with CKD who also experience multimorbidity and/or frailty. This study will explore these topics.

### Aims and objectives

The overall aim of this study is to explore patient and HCP perspectives and expectations of the use of KFRE in individuals with CKD and multimorbidity and/or frailty, with a focus on SDM.

The study objectives are as follows:

- To understand patient and HCP perceptions of kidney failure risk in the context of multimorbidity and frailty.
- To explore how patients with CKD and multimorbidity and/or frailty wish to discuss kidney failure risk and if this differs from HCP’s perspectives.
- To investigate patient and HCP expectations about how the KFRE should be used in the care and decision-making process of individuals with multimorbidity and/or frailty.
- To identify what barriers and challenges exist in using the KFRE to guide treatment and referral in individuals with multimorbidity and/or frailty.

## Methods and analysis

### Study design

Semistructured interviews and focus groups will be conducted and analysed to explore patient and HCP perspectives on kidney failure risk and use of the KFRE in individuals with multimorbidity and/or frailty.

The study will involve framework analysis^26 27^ of in-depth individual patient interviews and HCP focus groups. Patients and HCPs are being recruited to allow an exploration of the topic of SDM. Individual interviews for patients are being used to allow exploration of the potentially distressing subject of kidney failure risk and to provide insights into personal feelings and perspectives on this topic. Interviews were also chosen for patients to allow flexibility and enhance ability to participate as we recognise that some individuals may find participating in focus groups challenging or burdensome. Focus groups are being used for HCP participants to capture a breadth of perspectives and build on interactions that exist within multidisciplinary teams.

### Study setting and participant recruitment

Purposeful sampling will be used to recruit participants with experiences relevant to the research aims and objectives and to gain a variation in characteristics such as age, gender, ethnicity and healthcare role.^28 29^

Scottish primary care practices and secondary care kidney hospital departments in the Greater Glasgow and Clyde (GGC) Health Board, which provide renal services to a population of approximately 1.2 million, will be approached to recruit patients with CKD and multimorbidity and/or frailty and HCPs.

Potential participants from primary care will be identified through the National Health Service Research Scotland Primary Care Research Network (NRS PCRN), which will recruit through National Health Service (NHS) primary care practices. We will also recruit through SHARE (The Scottish Health Research Register and Biobank) who have a list of individuals registered with primary care practices across Scotland who are willing to be contacted to participate in research. Participants will be identified through an IT system search of primary care records (identified via CKD coding and long-term condition and frailty coding where available), with subsequent screening by the patient’s own GP and the research team. Potential participants will be sent written information and an invitation letter by post.

Patients from secondary care kidney clinics will be screened by their named HCP to ensure they meet the inclusion criteria and are willing to be approached to participate in the study. These potential participants will then be approached by the research team via telephone or when attending clinic, to discuss the research project. Written information and an invitation letter will be provided in person, by email or by post.

Potential participants will also be approached through social media platforms, third-sector organisations and patient and public involvement and engagement (PPIE) groups. They will be invited to contact the research team directly or return an expression of interest form.

**Figure 1:**
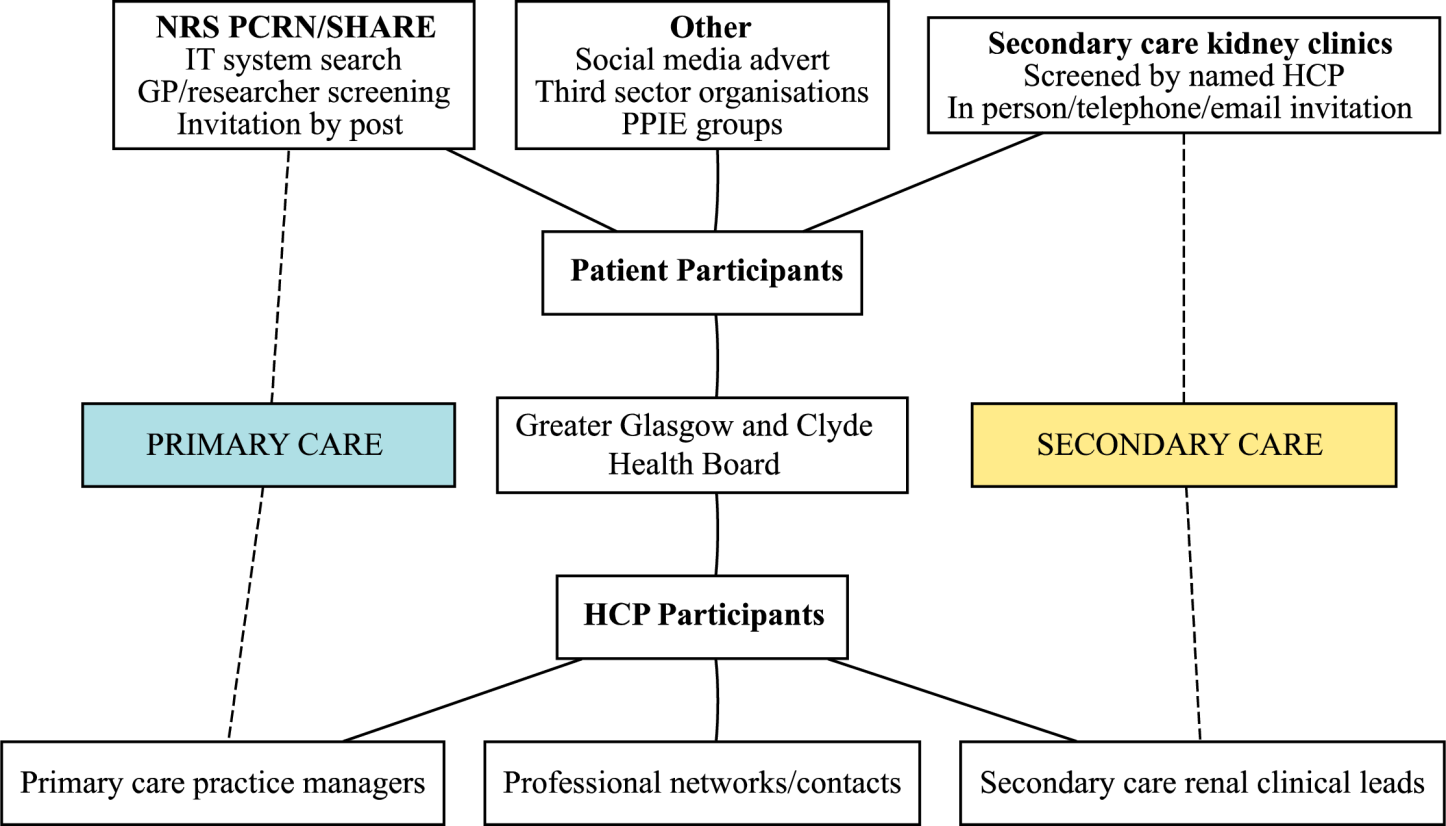
Participant recruitment strategy diagram. HCP, healthcare professional; NRS PCRN, National Health Service Scotland Primary Care Research Network; PPIE, patient and public involvement and engagement; SHARE, The Scottish Health Research Register and Biobank.

HCPs involved in discussing kidney disease and risk of kidney failure with patients will be identified by practice managers (primary care), renal clinical leads (secondary care) and professional contacts and networks (both primary and secondary care). HCPs will include doctors (specialty registrars (doctors in renal specialty training), specialty doctors (doctors who have chosen to work in renal but do not hold a training or consultant post), consultants and GPs) and specialist nurses. Clinical staff identified will then be screened by the research team to ensure they meet the inclusion criteria. All potentially suitable individuals will be contacted via email with a participant information sheet and invitation attached. An overview of the participant recruitment strategy is summarised in figure 1.

Once potential participants have expressed an interest, a researcher will contact them to arrange a suitable time for interview/focus group.

### Inclusion and exclusion criteria

Study inclusion and exclusion criteria are described in table 1.

**Table 1:** Participant inclusion and exclusion criteria

	Patient participants	Healthcare professional participants
Inclusion criteria	Adults (aged ≥18 years) with a diagnosis of CKD (defined as eGFR<60 mL/min/1.73 m^2^) and multiple long-term conditions (2 or more) or frailty defined by primary care read codes. Able to read and communicate in English. Able to provide informed consent.	Involved in the care of patients with CKD and explaining risk of kidney failure. Able to provide informed consent.
Exclusion criteria	Children and young people aged <18 years. Individuals already established on chronic kidney replacement therapy, in the form of dialysis or a transplant. Patients approaching end of life. Unable to give informed consent. Unable to read or communicate in English.	Not involved in the care of patients with CKD. Unable to provide informed consent.

CKDchronic kidney diseaseeGFRestimated glomerular filtration rate

### Study sample size

The aim is to recruit approximately 30 patient participants in total (20 from primary care and 10 from secondary care) to allow adequate sampling for saturation of themes. This split will allow us to explore the perspectives of both individuals who have CKD but may not have discussed kidney failure or attended specialist assessment and individuals who have experienced this process.

A mixture of approximately 20 primary and secondary care HCPs will be recruited to participate in a focus group. Focus groups will include around five individuals each and will be run as separate focus groups for primary and secondary care HCPs to explore if there are differences in the perspectives of these populations.

Sample sizes are appropriate to the study design, as qualitative research prioritises covering a range of views and contexts relevant to the topic rather than achieving representativeness. Sample sizes have been informed by previous qualitative research in similar populations/areas.^17 30 31^

### Consent

Participants will be sent a participant information sheet and consent documents prior to participating in their interview/focus group session. Individuals will be able to contact the research team directly (by email or telephone) if they wish to ask questions and will be given an opportunity to discuss the study further at the time of the interview/focus group. Written informed consent will be obtained from participants immediately prior to the interview/focus group.

### Data collection

Interview and focus group schedules will be developed based on the research questions, the Ottawa Decision Support Framework (ODSF) and the Ottawa Personal Decision Guide^32^ (OPDG) (a decision aid developed to help individuals make decisions and facilitate SDM based on the ODSF).^33^ The interview and focus group questions/guides were constructed based on the components of ODSF and OPDG (knowledge, values, support, certainty and decision-making). The ODSF and OPDGs were chosen over other SDM frameworks and guides due to their established use across a range of health decisions.^33^ This enables the adaptation of their themes for use in the context of SDM relating to risk of kidney failure and the use of the KFRE to guide care. Additionally, their use ensures important topics relevant to individuals making health-related decisions are captured within the study. ODSF was chosen above other SDM frameworks.

Although the study has a focus on SDM, the study also aims to explore individuals’ perceptions of kidney failure risk in the context of multimorbidity and frailty, communication of kidney failure risk, the use of KFRE in the care/treatment of individuals with multimorbidity and/or frailty and what barriers and challenges exist in using the KFRE in clinical practice. Patient participant interviews and HCP focus group guides are provided in online supplemental Text 1 and 2.

At the time of interviews and focus groups, participants will be asked to provide basic demographic information including age, gender, ethnic group, postcode (to allow Scottish Index of Multiple Deprivation (SIMD) decile to be determined) and their main healthcare setting experience. Additionally, patient participants will be asked about long-term conditions that they currently have, medications they are prescribed and to complete, with assistance from the interviewer, a clinical frailty scale as measurements of multimorbidity and frailty.

Interviews and focus groups will be performed/facilitated by one member of the research team (HW). Interviews will take place in the participants’ homes, in a secondary care clinic room or online via Zoom video conferencing software, depending on participant preference. Focus groups will take place in a hospital or GP practice meeting room or online, dependent on suitability/participant preference. Interviews will be offered to those who cannot attend focus groups. Interviews and focus groups will be digitally recorded and transcribed verbatim.

### Data analysis

Data collection has been developed around the components of the ODSF. An alternative framework has been chosen for data analysis. Framework analysis,^26 27^ underpinned by normalisation process theory (NPT),^34 35^ will be used to develop codes and explore themes from the interview and focus group transcripts. The five stages of framework analysis, as described by Ritchie and Lewis, will be followed: familiarisation, identifying a thematic framework, indexing, charting, mapping and interpretation.^26 27^ NPT is a middle-range theory^36 37^ that has been developed to understand the implementation, organisation and utilisation of processes and practices that are embedded, integrated and sustained in day-to-day life.^38^ The theory is divided into four mechanisms: coherence (making sense of processes), cognitive participation (engagement, organisation and commitment of individuals to tasks), collective actions (enablement of efforts and resources for practices including doing tasks and communicating knowledge) and reflexive monitoring (appraisal of cost and benefits of tasks and reorganising based on this evaluation).^34^ It has been used to understand the implementation of healthcare interventions from an HCP perspective,^39^ the embedding of treatments in the everyday life of a patient^40^ and to understand the treatment burden and the burden of self-care practices in patients with chronic diseases.^40^ NPT, therefore, provides a way of exploring and understanding the social processes that allow new models of care to be incorporated in healthcare settings. Self-care practices and SDM have similarities, for example, both involve patient roles/responsibilities and interactions with HCPs. We propose that NPT is a useful framework to aid understanding of how SDM is implemented and embedded in a patient’s life and health journey, and how HCPs implement and sustain a new process (here KFRE) in the SDM care of patients.

The analysis will primarily be conducted by the lead researcher on the project (HW), and 10% of transcripts will be check-coded by a second individual (KIG) to reduce unconscious bias within the analysis.

The analysis will be performed by using NVivo data analysis (V.14 (QSR International (UK))).

### Ethics and dissemination

Ethical approval was granted in August 2023 by the NHS Health Research Authority following a favourable opinion from the West if Scotland Research Ethics Committee (REC) 3 (IRASID: 325848, REC reference: 23WS/0119, Protocol number GN22RE559).

There is a possibility that individuals recruited from primary care may be unaware of their CKD diagnosis prior to recruitment, making this information new to them. This could be viewed as both a potential risk and a benefit. Individuals taking part in the research will be offered the chance to have their kidney failure risk score calculated and discussed with them. Discussing the possibility of worsening kidney function could be difficult and this topic will be approached sensitively, with the option to withdraw from the study if desired, and encouragement to speak to their usual health professional if upset occurs. Distress protocols for participants and researchers have been developed in case any distress related to the study occurs.

The findings of the study will be reported following the Consolidated criteria for Reporting Qualitative Research checklist.^41^

Findings will be published in a peer-reviewed, open-access journal, in line with Wellcome’s research output guidance—to allow work to be accessed free of cost and without restriction. Permission will be sought on consent forms for future use of fully anonymised data for the purposes of teaching or research (secondary analysis) for up to 10 years after the study has ended.

Findings will be disseminated to academic colleagues through presentations at national and international conferences.

Results from the study will be fed back to PPIE groups that have been involved in the study, prior to and following publication. Results will also be feedback to participating GP practices via NRS PCRN and included in SHARE newsletters. A lay report will be produced and disseminated through the third-sector and social media. To share information and exchange the results of the study, a knowledge exchange event for participants, patients, carers and members of the public following the completion of the project is planned. Kidney research charities and community groups, as well as our PPIE groups, will be approached to help advertise and widen the reach of our knowledge exchange event.

Dissemination to clinicians will be facilitated through a national KFRE implementation group and sharing of findings with clinical guidance policy makers such an NICE and KDIGO.

### Equity of access to the study

This study is being conducted in GGC, Scotland. The location provides diversity in ethnicity and socioeconomic deprivation.^42^ Efforts will be made to recruit individuals proportionate to the background demographics of the Scottish population (based on age, gender, ethnic group and SIMD) where possible within the constraints of time and resources of the study.

### Patient and public involvement and engagement

The study development has been informed by six people with lived experience of CKD (the University of Glasgow West of Scotland Kidney PPIE group). They will continue to identify the key areas they feel PPIE would be useful, advise on recruitment of participants and aid dissemination of results with themselves and the wider public.

The study has had further engagement with a similar sized group of patients who do not all specifically have experience of kidney disease (MVLS PPIE group), as we acknowledge the views and ideas of people who have not yet experienced kidney failure might be different to those who have.

The West of Scotland Kidney PPIE group contributed to the development and refinement of participant-facing materials, including participant information sheets, consent forms and interview schedules.

There is future planned involvement for the above PPIE groups in reviewing themes that are identified from the thematic analysis.

### Strengths and limitations

This study is designed to explore the understanding of patient and HCP perspectives on kidney failure risk in the context of multimorbidity and/or frailty and how KFRE might be used in SDM. The resulting data and recommendations will be dependent on individuals who participate in the interviews and focus group sessions. The study aims to recruit patients and HCPs from both primary and secondary care, to minimise bias and increase the generalisability of results. However, participants will be recruited from a single region in Scotland, UK, which may limit the diversity of the study participants. Additionally, individuals approaching the end of life, those unable to provide informed consent and those who cannot speak English are excluded from participating in the study, limiting the validation of findings in these populations.

A key strength of this study is that all aspects have been developed in partnership with the patient and public representatives. The study concept and aims and study materials have been reviewed by PPIE groups, as already described. This has ensured that the study has a more patient-centred focus and that participant-facing materials are understandable and accessible to participants.

Underpinning data analysis with middle-range theory will provide strength in informing how SDM can be used in individuals with CKD also experiencing multimorbidity or frailty to explore kidney failure risk.

### Impact

We anticipate that the findings from this project have the potential to influence clinical practice, guidelines and policy in relation to the management of patients with CKD with the added complexities of multimorbidity and/or frailty. In particular, findings will have the potential to promote a more holistic and comprehensive approach and provide an understanding of how best to explore and undertake SDM around kidney failure risk with this subgroup of patients. The study will provide insights into how KFRE or similar prediction tools might be used or impact these individuals and their care.

## Conclusion

This qualitative study will explore patient and HCP perspectives on kidney failure risk and the use of KFRE in individuals with CKD and multimorbidity and/or frailty. Framework analysis, underpinned by NPT, will guide themes and support recommendations from the study. This under-researched topic is key for guiding HCPs, policy and clinical guidance for this group of patients.

## supplementary material

10.1136/bmjopen-2024-085843 online supplemental file 1

10.1136/bmjopen-2024-085843 online supplemental file 2

## References

[1] MacRae C, Mercer SW, Guthrie B (2021). Comorbidity in chronic kidney disease: a large cross-sectional study of prevalence in Scottish primary care. *Br J Gen Pract*.

[2] Hanlon P, Nicholl BI, Jani BD (2018). Frailty and pre-frailty in middleaged and older adults and its association with multimorbidity and mortality: a prospective analysis of 493 737 UK Biobank participants. Lancet Public Health.

[3] Fried LP, Tangen CM, Walston J (2001). Frailty in older adults: evidence for a phenotype. J Gerontol A Biol Sci Med Sci.

[4] Morley JE, Vellas B, van Kan GA (2013). Frailty consensus: a call to action. J Am Med Dir Assoc.

[5] KDIGO (2013). KDIGO 2012 Clinical Practice Guideline for the Evaluation and Management of Chronic Kidney Disease.

[6] NICE (2021). Chronic kidney disease: assessment and management 2021 guideline.

[7] Tangri N, Stevens LA, Griffith J (2011). A predictive model for progression of chronic kidney disease to kidney failure. Jama.

[8] Sullivan MK, Jani BD, Rutherford E (2022). Potential impact of NICE guidelines on referrals from primary care to nephrology. Br J Gen Pract.

[9] Johnston MC, Black C, Mercer SW (2020). Prevalence of secondary care multimorbidity in mid-life and its association with premature mortality in a large longitudinal cohort study. BMJ Open.

[10] Smith SM, Wallace E, Clyne B (2021). Interventions for improving outcomes in patients with multimorbidity in primary care and community setting: a systematic review. Syst Rev.

[11] Bowling CB, Vandenberg AE, Phillips LS (2017). Older Patients’ Perspectives on Managing Complexity in CKD Self-Management. CJASN.

[12] Elwyn G, Frosch D, Thomson R (2012). Shared decision making: a model for clinical practice. J Gen Intern Med.

[13] Pel-Littel RE, Buurman BM, van de Pol MH (2023). Effects of a shared decision making intervention for older adults with multiple chronic conditions: the DICO study. BMC Med Inform Decis Mak.

[14] Pel-Littel RE, Snaterse M, Teppich NM (2021). Barriers and facilitators for shared decision making in older patients with multiple chronic conditions: a systematic review. BMC Geriatr.

[15] Cassidy BP, Getchell LE, Harwood L (2018). Barriers to Education and Shared Decision Making in the Chronic Kidney Disease Population: A Narrative Review. Can J Kidney Health Dis.

[16] House TR, Wightman A, Rosenberg AR (2022). Challenges to Shared Decision Making About Treatment of Advanced CKD: A Qualitative Study of Patients and Clinicians. Am J Kidney Dis.

[17] Morton RL, Tong A, Howard K (2010). The views of patients and carers in treatment decision making for chronic kidney disease: systematic review and thematic synthesis of qualitative studies. BMJ.

[18] Ho YF, Chen YC, Li IC (2021). A qualitative study on shared decision-making of patients with chronic kidney disease. Nurs Open.

[19] Engels N, de Graav GN, van der Nat P (2022). Shared decision-making in advanced kidney disease: a scoping review. BMJ Open.

[20] Yu X, Nakayama M, Wu M-S (2022). Shared Decision-Making for a Dialysis Modality. Kidney Int Rep.

[21] van der Horst DEM, Hofstra N, van Uden-Kraan CF (2023). Shared Decision Making in Health Care Visits for CKD: Patients’ Decisional Role Preferences and Experiences. Am J Kidney Dis.

[22] Smekal MD, Tam-Tham H, Finlay J (2018). Perceived Benefits and Challenges of a Risk-Based Approach to Multidisciplinary Chronic Kidney Disease Care: A Qualitative Descriptive Study. Can J Kidney Health Dis.

[23] Smekal MD, Tam-Tham H, Finlay J (2019). Patient and provider experience and perspectives of a risk-based approach to multidisciplinary chronic kidney disease care: a mixed methods study. BMC Nephrol.

[24] Bujold M, Pluye P, Légaré F (2022). Decision-making and related outcomes of patients with complex care needs in primary care settings: a systematic literature review with a case-based qualitative synthesis. *BMC Prim Care*.

[25] Brown EL, Poltawski L, Pitchforth E (2022). Shared decision making between older people with multimorbidity and GPs: a qualitative study. *Br J Gen Pract*.

[26] Gale NK, Heath G, Cameron E (2013). Using the framework method for the analysis of qualitative data in multi-disciplinary health research. BMC Med Res Methodol.

[27] Ritchie J, Lewis J, McNaughton NC (2003). Qualitative Research Practice: A Guide for Social Science Students and Researchers.

[28] Palinkas LA, Horwitz SM, Green CA (2015). Purposeful Sampling for Qualitative Data Collection and Analysis in Mixed Method Implementation Research. Adm Policy Ment Health.

[29] Renjith V, Yesodharan R, Noronha JA (2021). Qualitative Methods in Health Care Research. Int J Prev Med.

[30] Kahn LS, Vest BM, Madurai N (2015). Chronic kidney disease (CKD) treatment burden among low-income primary care patients. Chronic Illn.

[31] Lloyd A, Joseph-Williams N, Edwards A (2013). Patchy “coherence”: using normalization process theory to evaluate a multi-faceted shared decision making implementation program (MAGIC). Implement Sci.

[32] O’Connor SJ (2015). Ottawa personal decision guide: ottawa hospital research. https://decisionaid.ohri.ca/decguide.html.

[33] Hoefel L, Lewis KB, O’Connor A (2020). 20th Anniversary Update of the Ottawa Decision Support Framework: Part 2 Subanalysis of a Systematic Review of Patient Decision Aids. Med Decis Making.

[34] May C, Finch T (2009). Implementing, Embedding, and Integrating Practices: An Outline of Normalization Process Theory. Sociology.

[35] May CR, Mair F, Finch T (2009). Development of a theory of implementation and integration: Normalization Process Theory. Implement Sci.

[36] Bailey KD (1991). Alternative procedures for macrosociological theorizing. Qual Quant.

[37] Merton RK (1957). Social Theory and Social Structure.

[38] Gallacher K, Morrison D, Jani B (2013). Uncovering treatment burden as a key concept for stroke care: a systematic review of qualitative research. PLoS Med.

[39] Pope C, Halford S, Turnbull J (2013). Using computer decision support systems in NHS emergency and urgent care: ethnographic study using normalisation process theory. BMC Health Serv Res.

[40] Gallacher K, May CR, Montori VM (2011). Understanding patients’ experiences of treatment burden in chronic heart failure using normalization process theory. Ann Fam Med.

[41] Booth A, Hannes K, Harden A (2014). Guidelines for Reporting Health Research: A User’s Manual.

[42] Scottish government (2020). Scottish index of multiple deprivation. https://www2.gov.scot/Topics/Statistics/SIMD.

