# Supplementary material for: Exploration of patients’ and healthcare professionals’ perspectives on kidney failure risk and the use of the kidney failure risk equation in MULTIPle lOng-term condItions aNd frailTy (MULTIPOINT) study: a qualitative interview and focus group study protocol
Source: BMJ Open. 2024 Oct 18;14(10):e085843. doi: 10.1136/bmjopen-2024-085843 (PMC11492959; doi:10.1136/bmjopen-2024-085843)
Supplement: online supplemental file 1 [file bmjopen-14-10-s001.pdf]

Supplementary text 1. Patient participant interview schedule

INTERVIEW GUIDE: Kidney failure risk in multiple long-term conditions and frailty – exploration of patients' and healthcare professionals' perspectives of kidney failure risk

Background Information for the Interviewer

In situations where there is a lack of knowledge, questions will be posed in a manner which takes account of such a limitation.

The interview will be semi-structured in format, and thus the exact wording and prompts used may vary between patients to encourage the patient to share their views and allow them an opportunity to talk at length about their thoughts, ideas and feelings about their illnesses and shared decision making in healthcare.

Not all questions may be asked and participants we be able to guide the topics.

Introduction Procedure with Patients

1. Give complete name.
2. Identify self as a researcher from the Department of Cardiovascular and Metabolic Health at the University of Glasgow.
3. Give short explanation of the purpose of the study:

'I would like you to help me understand your thoughts and ideas around the risk of kidney failure in people with kidney disease, multiple long-term conditions, and frailty. I am interested in particular in how this topic should be explored and discussed between patients and healthcare professionals and what the expectations of using a tool called the Kidney Failure Risk Equation in the decision-making process and guiding care are.

The Kidney Failure Risk Equation or KFRE is a tool that uses an individual's age, sex, a blood test that measures kidney function called estimated glomerular filtration rate and a urine test that looks for protein to see how well the kidneys are working called urine albumin creatinine ratio to estimate the chance of their kidney problem progressing to kidney failure. The tool calculates the risk of kidney failure as a percentage risk over the next 5 years, so for example a 10% risk means that out of 100 people with that score, 10 will develop kidney failure over the next 5 years. Recent NICE guidelines have suggested that people who have

a more than 5% risk of kidney failure (as calculated using the KFRE tool) within 5 years should be referred to hospital kidney doctors.

Please feel assured that no one will be able to identify you from what you say when talking to me and everything you tell me will be treated in the strictest confidence.'

If at any time you want to stop, or have a break, please let me know.

'I will be recording the interview, so I can remember all that you have said to me.'

'Do you have any questions about the study or anything you would like me to explain further?'

PARTICIPANT PROFILE

Subject ID:

Age:

Gender:

- ☐ Male
- ☐ Female
- ☐ Non-binary
- ☐ Other
- ☐ Would rather not say

What is your ethnic group?

Choose one option that best describes your ethnic group or background

White

- ☐ Scottish
- ☐ Other British
- ☐ Irish
- ☐ Gypsy/Traveller
- ☐ Polish
- ☐ Any other White ethnic group, please describe

Mixed or Multiple ethnic groups

- ☐ Any Mixed or Multiple ethnic group, please describe

Asian, Asian Scottish or Asian British

- ☐ Pakistani, Pakistani Scottish or Pakistani British
- ☐ Indian, Indian Scottish or Indian British
- ☐ Bangladeshi, Bangladeshi Scottish or Bangladeshi British
- ☐ Chinese, Chinese Scottish or Chinese British
- ☐ Any other Asian group, please describe

African

- ☐ African, African Scottish or African British
- ☐ Any other African group, please describe

Caribbean or Black

- ☐ Caribbean, Caribbean Scottish or Caribbean British
- ☐ Black, Black Scottish or Black British
- ☐ Any other Caribbean or Black group, please describe

Other ethnic group

- ☐ Arab, Arab Scottish or Arab British
- ☐ Any other ethnic group, please describe

Main setting of current healthcare for kidney disease:

- ☐ Hospital Setting
- ☐ Community/GP practice
- ☐ Other

How long have you had kidney disease for? (Please provide as close an estimate as you can but don't worry if you don't know the exact date)

____

____

Please list any medical conditions that you have:

____

____

____

____

Please list any medications that you take:

____

____

____

____

<u>With the assistance of your interviewer please use the following scale to pick the best description of your current activity and functional status:</u>

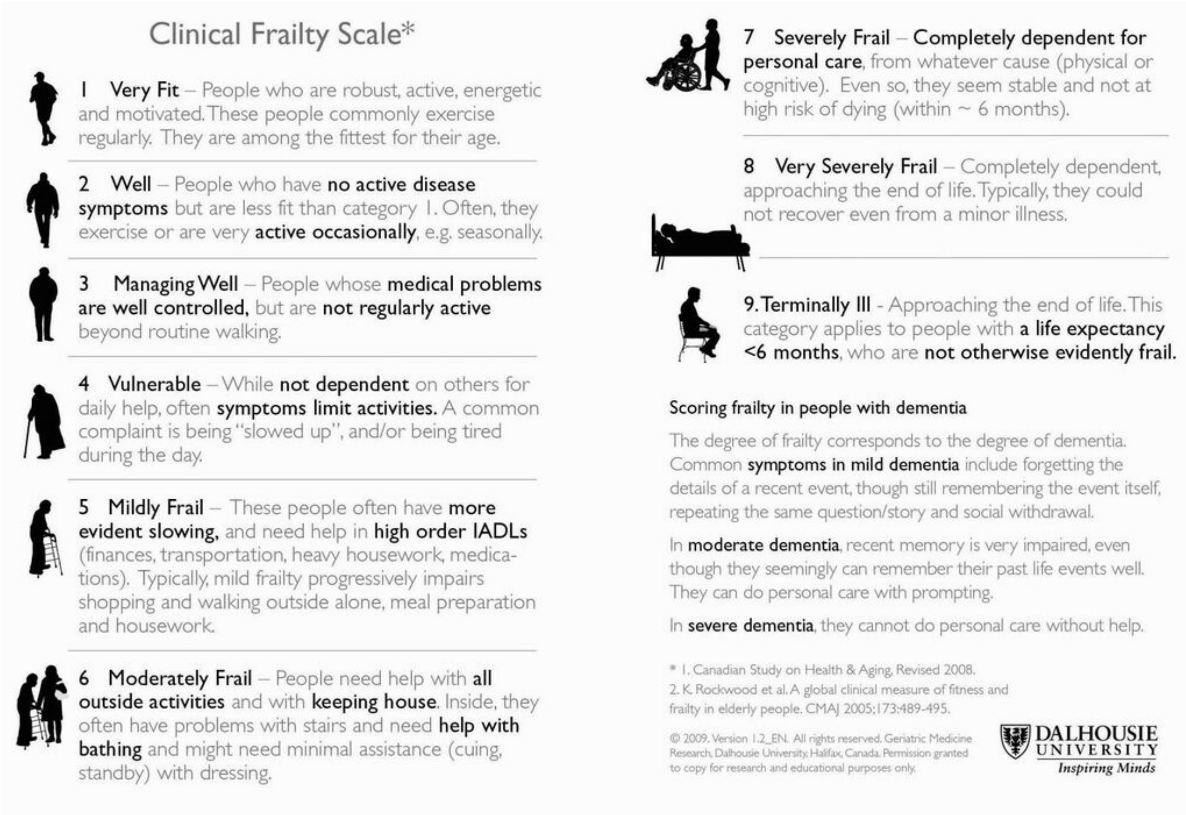

Clinical Frailty Scale*

1 **Very Fit** – People who are robust, active, energetic and motivated. These people commonly exercise regularly. They are among the fittest for their age.

2 **Well** – People who have **no active disease symptoms** but are less fit than category 1. Often, they exercise or are very **active occasionally**, e.g. seasonally.

3 **Managing Well** – People whose **medical problems are well controlled,** but are **not regularly active** beyond routine walking.

4 **Vulnerable** – While **not dependent** on others for daily help, often **symptoms limit activities.** A common complaint is being "slowed up", and/or being tired during the day.

5 **Mildly Frail** – These people often have **more evident slowing,** and need help in **high order IADLs** (finances, transportation, heavy housework, medications). Typically, mild frailty progressively impairs shopping and walking outside alone, meal preparation and housework.

6 **Moderately Frail** – People need help with **all outside activities** and with **keeping house**. Inside, they often have problems with stairs and need **help with bathing** and might need minimal assistance (cuing, standby) with dressing.

7 **Severely Frail** – **Completely dependent for personal care**, from whatever cause (physical or cognitive). Even so, they seem stable and not at high risk of dying (within ~ 6 months).

8 **Very Severely Frail** – Completely dependent, approaching the end of life. Typically, they could not recover even from a minor illness.

9. **Terminally Ill** - Approaching the end of life. This category applies to people with **a life expectancy <6 months**, who are **not otherwise evidently frail.**

Scoring frailty in people with dementia

The degree of frailty corresponds to the degree of dementia. Common **symptoms in mild dementia** include forgetting the details of a recent event, though still remembering the event itself, repeating the same question/story and social withdrawal.

In **moderate dementia**, recent memory is very impaired, even though they seemingly can remember their past life events well. They can do personal care with prompting.

In **severe dementia**, they cannot do personal care without help.

* 1. Canadian Study on Health & Aging, Revised 2008.
2. K. Rockwood et al. A global clinical measure of fitness and frailty in elderly people. CMAJ 2005;173:489-495.

© 2009. Version 1.2_EN. All rights reserved. Geriatric Medicine Research, Dalhousie University, Halifax, Canada. Permission granted to copy for research and educational purposes only.

DALHOUSIE UNIVERSITY *Inspiring Minds*

Date and Time of Interview:

The interviewer will explore the following general areas in an open fashion:

- **Knowledge**
- **Values**
- **Support**
- **Certainty**
- **Decision making**

Knowledge of kidney disease and kidney failure

What information do you feel is necessary to make decisions about the care you receive for your kidney disease?

What information do you feel is necessary for you to understand your risk of kidney failure (where treatments are required to do the job the kidneys would usually do (dialysis) or a kidney donated from someone else)?

Do you feel that information provision is currently adequate?

If not, how could it improve?

What facilitates and prevents good information provision?

How do you wish to discuss or explore your risk of kidney failure?

Do you feel this differs to your healthcare professional's perspectives?

If so how?

Knowledge of multimorbidity and frailty

What do you feel the impact of having kidney disease and other long term medical conditions or being frail is on your healthcare?

What information do you think you need to understand the needs or competing needs/risks of your multiple long-term conditions and kidney disease?

Do you think that other long term health conditions or frailty are discussed adequately alongside your kidney disease?

If not, how could this be improved?

Who should discuss other long term health conditions alongside kidney disease e.g. hospital kidney doctors/specialist kidney nurses / primary care GP or nurse/mixture?

Communication

How well do hospital teams and/or primary care teams communicate with and to you?

What facilitates and prevents the above communication?

Priorities/key values

What matters to you most in relation to your kidney disease and other long-term conditions/frailty?

What risks and benefits or pros and cons are important to you?

Support

What support do you have or feel you need to make decisions about your healthcare?

Who do you discuss your health and care and any related decisions with?

Do you think an individual's social network (friends, family, neighbours) influence their role in shared decision making (making health decisions with health professionals)?

If so, how?

Uncertainty

How certain do you feel about the choices you make in relation to your kidney disease and other long-term conditions?

Are there any areas you feel uncertain about?

If so, how do you manage these uncertainties?

Do you discuss uncertainties with your healthcare professional?

Do you think you would be more or less likely to want to be referred to hospital kidney doctors/discuss your risk of kidney failure if you have reached a point in life when you have lots of health conditions or are frail?

Choices/decisions

How do you think the kidney failure risk equation (a tool that can estimate the chance of someone's kidney problem progressing to kidney failure) should be used in the care of individuals with multiple long-term conditions and/or frailty?

How do you think the kidney failure risk equation (a tool that can estimate the chance of someone's kidney problem progressing to kidney failure) should be used in the decision-making process of individuals with multiple long-term conditions and/or frailty?

How do you think the decision to refer people with multiple long-term conditions/frailty and kidney disease to hospital kidney doctors should be made? Should it involve the kidney failure risk equation and if so how?

What facilitates and prevents the use of the Kidney Failure Risk Equation being used to guide care/treatment/referral to hospital kidney doctors?

Shared decision making

What role do you prefer to take in making choices or decisions related to your health and healthcare?

Who do you think should make decisions?

How often should these decisions be reviewed and by whom?

Do you think that shared decision making is helpful to people with kidney disease and multiple long-term conditions?

Do you think that shared decision making (making health decisions with health professionals) is helpful to people with kidney disease and frailty?

What do you think facilitates and prevents shared decision making?

What are your thoughts on decision aids/tools that might help you make decisions?

At close of interview

The interviewer will ask the participant if there are any issues they would like to mention which haven’t been covered.

Thank the participant and reiterate that all they have discussed is confidential.

Supplementary text 2. Healthcare professional participant focus group schedule

<u>FOCUS GROUP GUIDE:</u> Kidney failure risk in multiple long-term conditions and frailty – exploration of patients' and healthcare professionals' perspectives of kidney failure risk

Background Information for the Interviewer

In situations where there is a lack of knowledge, questions will be posed in a manner which takes account of such a limitation.

The focus group will be semi-structured in format, and thus the exact wording and prompts used may vary between groups to encourage individuals to share their views and allow them an opportunity to talk at length about their thoughts, ideas and feelings about the Kidney Failure Risk Equation and multimorbidity and shared-decision making in healthcare.

Not all questions may be asked and participants we be able to guide the topics.

Introduction Procedure with Participants

1. Give complete name.
2. Identify self as a researcher from the Department of Cardiovascular and Metabolic Health at the University of Glasgow.
3. Give short explanation of the purpose of the study:

'I would like you to help me understand your thoughts and ideas around the risk of kidney failure in people with kidney disease, multiple long-term conditions/multimorbidity and frailty. I am interested in particular in how this topic should be explored and discussed between patients and healthcare professionals and what the expectations of using a tool called the Kidney Failure Risk Equation in the decision-making process and guiding care are.

The Kidney Failure Risk Equation or KFRE is a tool that uses an individual's age, sex, a blood test that measures kidney function called estimated glomerular filtration rate and a urine test that looks for protein to see how well the kidneys are working called urine albumin creatinine ratio to estimate the chance of their kidney problem progressing to kidney failure. The tool calculates the risk of kidney failure as a percentage risk over the next 5 years, so for example a 10% risk means that out of 100 people with that score, 10 will develop kidney failure over the next 5 years. Recent NICE guidelines have suggested that people who have

a more than 5% risk of kidney failure (as calculated using the KFRE tool) within 5 years should be referred to hospital kidney doctors.

Please feel assured that no one will be able to identify you from what you say when talking to me and everything you tell me will be treated in the strictest confidence.'

'If at any time you want to stop, or have a break, please let me know.'

'I will be recording the focus group, so I can remember everything that has been discussed.'

'Do you have any questions about the study or anything you would like me to explain further?'

PARTICIPANT PROFILE

Subject ID:

Age:

Gender:

- ☐ Male
- ☐ Female
- ☐ Non-binary
- ☐ Other
- ☐ Would rather not say

What is your ethnic group?

Choose one option that best describes your ethnic group or background

White

- ☐ Scottish
- ☐ Other British
- ☐ Irish
- ☐ Gypsy/Traveller
- ☐ Polish
- ☐ Any other White ethnic group, please describe

Mixed or Multiple ethnic groups

- ☐ Any Mixed or Multiple ethnic group, please describe

Asian, Asian Scottish or Asian British

- ☐ Pakistani, Pakistani Scottish or Pakistani British
- ☐ Indian, Indian Scottish or Indian British
- ☐ Bangladeshi, Bangladeshi Scottish or Bangladeshi British
- ☐ Chinese, Chinese Scottish or Chinese British
- ☐ Any other Asian group, please describe

African

- ☐ African, African Scottish or African British
- ☐ Any other African group, please describe

Caribbean or Black

- Caribbean, Caribbean Scottish or Caribbean British
- Black, Black Scottish or Black British
- Any other Caribbean or Black group, please describe

Other ethnic group

- Arab, Arab Scottish or Arab British
- Any other ethnic group, please describe

Profession:

- Renal consultant
- Renal doctor in training
- Renal specialist nurse
- GP
- Practice nurse
- Other - Please state:

Do you work in the:

- Hospital Setting
- Primary Care
- Other

How long have you worked in this job?

- <1 year
- 1-5 years
- 5-10 years
- >10 years

Please describe your role in caring for and discussing risks of kidney failure to patients with CKD:

____

____

Date and Time of Focus Group

The facilitator will explore the following general areas in an open fashion:

- **Knowledge**
- **Values**
- **Support**
- **Certainty**
- **Decision making**

Knowledge of kidney disease and kidney failure

What information do you feel patients require about chronic kidney disease?

What information do you feel patients require about their risk of kidney failure?

Do you feel that information provision is currently adequate?

If not, how could it improve?

What facilitates and prevents good information provision?

How and when should the risk of kidney failure be discussed or explored with patients?

Knowledge of multimorbidity and frailty

What are your perceptions of the risk of kidney failure in patients who have CKD, multimorbidity and/or frailty?

What needs or competing needs/risks do you think these patients should be informed of?

Who should discuss other long term health conditions alongside kidney disease e.g. hospital doctors/specialist kidney nurses /primary care GP or nurse/mixture?

Communication

How well do you feel you communicate about kidney disease and kidney failure risk with these types of patients (multimorbidity/frailty and CKD)?

What facilitates and prevents the above communication?

Priorities/key values

What are your key priorities in caring for these patients?

What do you think matters most to patients in relation to their kidney disease and other long-term conditions/frailty?

How do you explore this with patients and gather this information?

Support

What support do you have or feel you need to make decisions for these patients?

Does an individual's social network (friends, family, neighbours) influence their role in shared decision making?

If so, how?

Uncertainty

Are there areas of uncertainty in caring for and managing patients with CKD, multimorbidity and/or frailty?

If so, how do you manage these uncertainties?

Do you discuss these uncertainties with patients?

Do you think you would be more or less likely to refer patients to secondary care nephrology services/discuss their risk of kidney failure if they had multimorbidity and/or were frail?

Choices/decisions

How do you think the kidney failure risk equation (a tool that can estimate the chance of someone's CKD progressing to kidney failure) should be used in the care of individuals with multimorbidity and/or frailty?

How do you think the kidney failure risk equation (a tool that can estimate the chance of someone's CKD progressing to kidney failure) should be used in the decision-making process of individuals with multimorbidity and/or frailty?

How do you think the decision to refer people with multimorbidity and kidney disease to secondary care nephrology services should be made? Should it involve the kidney failure risk equation and if so how?

What facilitates and prevents the use of the Kidney Failure Risk Equation being used to guide care/treatment/referral to secondary care nephrology services?

Do you take into account an individual's multimorbidity/frailty when assessing their risk of kidney failure/referring to secondary care?

Shared decision making

What role do you think patients should to take in making choices or decisions related to their health and healthcare?

Who do you think should make decisions?

When thinking specifically about referring to secondary care nephrology services/discussing risk of kidney failure: How often should these decisions be reviewed and by whom?

Do you think that shared decision making is helpful to people with kidney disease and multiple long-term conditions?

Do you think that shared decision making is helpful to people with kidney disease and frailty?

What do you think facilitates and prevents shared decision making?

What are your thoughts on decision aids/tools that might help patients make decisions?

At close of focus group

The facilitator will ask the participant if there are any issues they would like to mention which haven't been covered.

Thank the participants and reiterate that all they have discussed is confidential.
