# Supplementary material for: Exploration of patients’ and healthcare professionals’ perspectives on kidney failure risk and the use of the kidney failure risk equation in MULTIPle lOng-term condItions aNd frailTy (MULTIPOINT) study: a qualitative interview and focus group study protocol
Source: BMJ Open. 2024 Oct 18;14(10):e085843. doi: 10.1136/bmjopen-2024-085843 (PMC11492959; doi:10.1136/bmjopen-2024-085843)
Supplement: online supplemental file 2 [file bmjopen-14-10-s002.docx]

# Note from the Editors: Instructions for reviewers of study protocols

Since launching in 2011, BMJ Open has published study protocols for planned or ongoing research studies. If data collection is complete, we will not consider the manuscript.

Publishing study protocols enables researchers and funding bodies to stay up to date in their fields by providing exposure to research activity that may not otherwise be widely publicised. This can help prevent unnecessary duplication of work and will hopefully enable collaboration. Publishing protocols in full also makes available more information than is currently required by trial registries and increases transparency, making it easier for others (editors, reviewers and readers) to see and understand any deviations from the protocol that occur during the conduct of the study.

The scientific integrity and the credibility of the study data depend substantially on the study design and methodology, which is why the study protocol requires a thorough peer-review.

*BMJ Open*will consider for publication protocols for any study design, including observational studies and systematic reviews.

Some things to keep in mind when reviewing the study protocol:

- Protocol papers should report planned or ongoing studies. The dates of the study should be included in the manuscript.
- Unfortunately we are unable to customize the reviewer report form for study protocols. As such, some of the items (i.e., those pertaining to results) on the form should be scores as Not Applicable (N/A).
- While some baseline data can be presented, there should be no results or conclusions present in the study protocol.
- For studies that are ongoing, it is generally the case that very few changes can be made to the methodology. As such, requests for revisions are generally clarifications for the rationale or details relating to the methods. If there is a major flaw in the study that would prevent a sound interpretation of the data, we would expect the study protocol to be rejected.
